# Application of renal-rotation techniques in retroperitoneoscopic partial nephrectomy

**DOI:** 10.3892/etm.2015.2294

**Published:** 2015-02-13

**Authors:** CHUN-HUA LIN, QING-ZUO LIU, KE WANG, SHENG-QIANG YU, HONG-WEI ZHAO, JIAN-TAO WANG, GUANG-LEI LI, ZHEN-LI GAO

**Affiliations:** Department of Urology, Affiliated Yantai Yuhuangding Hospital of Qingdao University Medical College, Yantai, Shandong 264000, P.R. China

**Keywords:** renal rotation, retroperitoneoscopy, partial nephrectomy

## Abstract

Retroperitoneoscopic partial nephrectomy (RPN) is one of the standard methods for treating T1-stage renal carcinoma, which has a narrow operational space and a difficult surgical procedure. The aim of this study was to examine the safety and feasibility of renal-rotation techniques in RPN. Between April 2012 and June 2014, the renal-rotation technique in RPN was performed in 22 male and 16 female patients, aged between 31 and 75 years (mean, 52 years), with stage T1N0M0 renal-cell carcinoma. In 29 cases the tumor was located at the ventral side of the kidney, including 22 cases at the renal hilum, and in nine cases the tumor was located at the inferior pole of the kidney. The tumor size was between 1.5 and 4.6 cm (mean, 2.8 cm). The results showed that, in all 38 cases, the procedure was successfully accomplished without conversion to open surgery. There were no intraoperative complications and only three cases of postoperative complications. The surgery duration was between 45 and 116 min (mean, 59 min); blood loss was between 10 and 120 ml (mean, 40 ml) and no patients required a blood transfusion. The average kidney ischemia time was 21 min (range, 15–38 min). No patients had local recurrence or metastasis after follow-up of between one and 26 months. In conclusion, the application of the renal-rotation technique in RPN for tumors located at the ventral side, renal hilum or at the inferior pole of the kidney is safe and feasible and worth wider clinical application.

## Introduction

For the effective treatment of localized renal carcinoma, surgical resection is considered the primary choice. Partial or radical nephrectomy may be used, depending on the tumor and the specific patient characteristics (1). Nephron-sparing partial nephrectomy is used when the tumor is small (<4 cm in diameter) or when the patient has other medical concerns, such as diabetes or hypertension (2). Radical nephrectomy is most often used when there is a large tumor present in only one kidney and the other kidney is fully functional. Open surgery is the traditional method for nephrectomy; however, with the development of technology and improvement in surgical skills, laparoscopic or robotic techniques are increasingly being used. According to the 2009 American Urological Association management guidelines, in patients with a T1 renal mass, complete surgical excision by partial nephrectomy is the standard treatment option and is strongly recommended (3). As compared with open partial nephrectomy, laparoscopic partial nephrectomy is technically challenging and associated with longer warm renal ischemia time, more major intraoperative complications and more postoperative urological complications (4,5). However, laparoscopic partial nephrectomy is minimally invasive, and achieves similar intermediate-term cancer cure and renal functional outcome, with the additional advantages of decreased postoperative narcotic use, earlier hospital discharge and a more rapid convalescence (5,6). With the development of laparoscopy and the widespread use of imaging technology, laparoscopic partial nephrectomy has become the standard for treating T1-stage renal carcinoma (6). Laparoscopic partial nephrectomy can be performed transperitoneally or retroperitoneally. Retroperitoneoscopic partial nephrectomy (RPN) has the advantages of minimal abdominal interference and convenience in processing renal pedicle vessels but it also bears drawbacks, including a narrow operational space, a lack of anatomical landmarks and a complex surgical procedure (7). The surgery is more difficult for tumors located at the ventral side of the kidney or at the inferior pole of the kidney, particularly at the renal hilum. Between April 2012 and June 2014, the renal-rotation technique was applied in the RPN surgery of 38 patients, in which the kidney was rotated at a certain angle following occlusion of the renal artery, prior to tumor excision and kidney suturing.

## Materials and methods

### Patients

**Figure 1:**
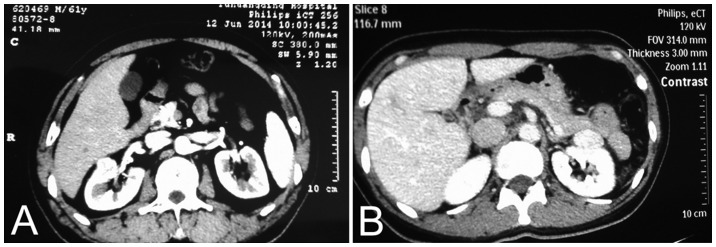
Renal computed tomography scans showing tumors located at the (A) right renal hilum and (B) left renal hilum.

The present study enrolled 38 patients, including 22 males and 16 females (age range, 31–75 years; mean, 52 years). The patients were confirmed as having renal carcinoma with no lymph node or renal vessel involvement by Color Doppler ultrasound, renal computed tomography (CT) or magnetic resonance imaging examination. The renal tumors were all at the T1N0M0 stage according to the American Joint Committee on Cancer TNM staging (8). The tumors were located at the ventral side of the kidney in 29 cases (including 22 at the renal hilum; Fig. 1) and at the inferior pole in nine cases. The diameter of the tumors ranged between 1.5 and 4.6 cm (mean, 2.8 cm). The tumors were confined to one kidney. No suspicious nodules were identified by thoracic CT. Levels of serum creatinine, blood urea nitrogen, blood sedimentation and alkaline phosphatase were relatively normal and there were no surgical contraindications. The present study was approved by the Medical Ethics Committee of the China Yantai Yuhuangding Hospital (Yantai, China). Written informed consent was obtained from either the patient or the patient’s family prior to involvement in the study and usage of the information for publication purposes.

### Surgical procedure

**Figure 2:**
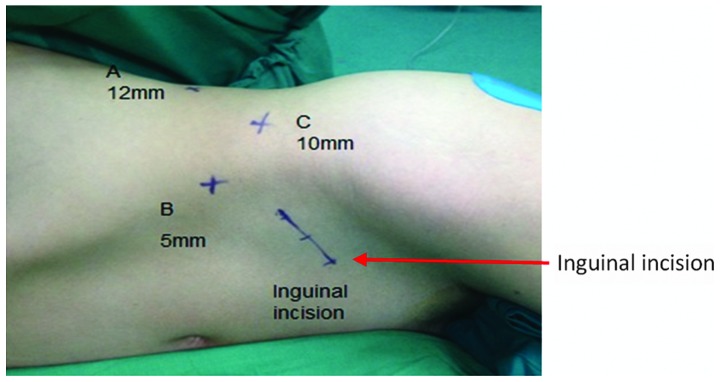
Location of the trocars and the inguinal incision. (A-C) Points of insertion for the (A) 12-mm, (B) 5-mm and (C) 10-mm trocars.

Following tracheal intubation anesthesia, patients adopted a 90° lateral position with a raised waist bridge. The skin was incised at the posterior axillary line 1 cm under the costal margin (Fig. 2, point A). The muscular layer and lumbar dorsal fascia were bluntly separated with long vascular forceps. The inside of the frame was examined with an index finger to confirm the entrance of the retroperitoneum and separation of the posterior peritoneum prior to imbedding a home-made latex balloon, inflated at 500–800 ml, for 3 min. Under guidance of the index finger, a puncture was made 2 cm above the axillary midline iliac crest (Fig. 2, point B) and 1 cm below the axillary front costal margin (Fig. 2, point C). Trocars of 12, 5 and 10 mm were inserted at points A, B and C, respectively. Subsequent to the closure of the incisions by suturing, a 0° or 30° laparoscope was fixed in trocar B and the main surgical equipment was inserted in trocar A. CO_2_ pneumoperitoneum was established and the pressure was maintained at 12–15 mmHg.

Subsequent to entering the retroperitoneum, the fatty tissue along the peritoneum and Gerota’s fascia was cleared from top to bottom and from front to back using a harmonic scalpel (Ethicon Endo-Surgery LLC, Guaynabo, Puerto Rico, US), so that the peritoneal reflection and Gerota’s fascia could be clearly identified. Gerota’s fascia was then incised near the peritoneal reflection, from over the upper pole of the kidney to 2–3 cm below the lower pole of the kidney, and as much as possible of the perirenal fascia and fatty tissue that obstructed the surgical field was removed forming an ‘arch window’ field for the convenience of the surgery.

Dissociation along the psoas major fascia exposed the renal pedicle, allowing separation of the renal artery. If accompanied by lumbar veins riding across the renal artery, the lumbar veins were disconnected using Hem-o-lok ligation clips (Teleflex Medical, Durham, NC, USA) prior to the complete dissociation of the renal artery. The perirenal adipose layer was then incised longitudinally along the outer renal edge and the kidney was dissociated completely from the fat layer. The renal week fat layer was removed, only retaining the renal hilum blood vessels and the renal collecting system fatty tissue.

**Figure 3:**
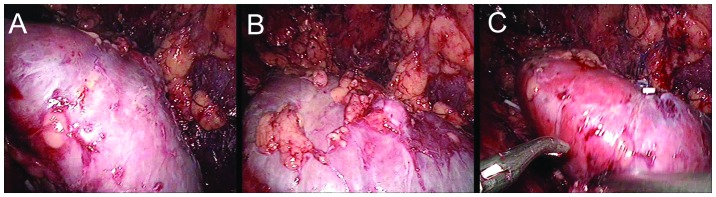
Positions of the kidney (A) prior to renal rotation; (B) subsequent to renal rotation; and (C) restored to its original position following suturing.

The direction of kidney rotation was decided by the size and location of the tumor. For tumors located at the ventral upper pole or at the renal hilum, the kidney was rotated internally for 45–90° toward the inferior pole. Following tumor removal and suturing, the kidney was rotated back to its original position (Fig. 3). If the tumor was located at the ventral inferior pole, the kidney was rotated internally for 45–90° toward the upper pole. During surgery the renal blood vessels were closely monitored to avoid vascular tears. The rotation angle could be adjusted at any time during the surgery for better exposure of the tumor.

Following complete exposure of the tumor, a bulldog clamp was used and the cross-clamp time was recorded. The tumor and surrounding tissues were excised 0.5–1.0 cm from the tumor margin. Wounds were then sutured using a no. 1 14×14 cm 1/2 circle bidirectional barbed absorption line (Quill™ line; Angiotech Pharmaceuticals, Inc., Reading PA, USA) (9). If renal pelvis and calyx fissure existed, the needle was inserted from the surface of the kidney, continuously closing the fissure without tying a knot, and then pierced out to the surface, continuously suturing the kidney wound. Each stitch was tightened and either tied with a knot, closed with Hem-o-lok or cut off the line at both ends. The blood vessel clamps were then loosened to check for evident bleeding. The tumor body was taken out from expanded point A and sent for pathological examination. A retroperitoneal drainage tube was put in place, the trocars were removed and the wound was closed up.

### Data collection

The surgery duration, intraoperative blood loss, renal artery occlusion (ischemic) time, suture time, duration of hospital stay and post-operative follow-ups were recorded.

## Results

In the present study, the 38 cases of RPN were completed successfully without conversion to open surgery. In no case did intraoperative complications occur, such as injury of large vessels or adjacent viscera. Three cases had postoperative complications, including one case of hematuria and two cases of subcutaneous emphysema. The patient with hematuria recovered following bed rest. The surgery duration was between 45 and 116 min (mean, 59 min). Intraoperative blood loss was between 10 and 120 ml (mean, 40 ml) and without intraoperative blood transfusion. Renal artery occlusion time was between 15 and 38 min (mean, 21 min). Pathological examination reported 29 cases of renal clear cell carcinoma and one case of papillary carcinoma, all with a negative margin, and eight cases of renal angiomyolipoma. Patients were followed for between one and 26 months subsequent to surgery (mean, 17 months), and there were no tumor recurrences or distant metastases. Renal functions remained normal.

## Discussion

Laparoscopic partial nephrectomy can be performed by two routes: the transperitoneal route or the retroperitoneal route. Following a comparison between retroperitoneal and transperitoneal laparoscopic partial nephrectomy procedures, Marszalek *et al* (10) suggested that the retroperitoneal approach had more advantages regarding surgery duration, blood loss and surgical complications; however, this approach also has drawbacks, such as a narrow operational space and a lack of anatomical landmarks. If the tumor is located at the ventral side or at the inferior pole, RPN is problematic due to the entry route of the laparoscope, which is also the bottleneck of the majority of RPN procedures.

During our long-term clinical practice and technical exploration, we found that the renal-rotation technique could solve the problem associated with RPN by providing sufficient operational space. Tumors located at the ventral side, inferior pole or even at the renal hilum could be removed using this technique. This technique is clearly advantageous in several aspects.

Firstly, the renal-rotation technique could markedly reduce the surgical complexity and increase the success rate of RPN. The most difficult aspect of RPN lies in the exposure of the ventral side renal tumor and subsequent suturing. For tumors located in the renal hilum, RPN would be even more problematic. Using the rotation technique the ventral side and even the hilum of the kidney can be clearly exposed, thus countering the disadvantage of the entry route. This is likely to increase the success rate of the surgery, boost the self-confidence of the surgeon and shorten the training time for young endoscopic urology physicians.

Another challenge of RPN is insufficient exposure of the surgical field. A limited operational space in the initial stage makes the procedure difficult, and may even cause the surgery to fail. As renal rotation requires the complete dissociation of the kidney and renal week fatty tissue, the surgical field is, therefore, relatively large and anatomical landmarks are obvious. This could also significantly reduce damage to the inferior vena cava, duodenum, liver and other organs caused by an obstructed surgical field, and reduce the incidence of complications, such as rupture of the peritoneum.

Finally, another aim for RPN is to reduce the renal warm ischemia time. The suturing of kidney sections is an important step affecting the warm ischemia time, and is the most effective method to maintain renal stability and prevent urine leakage. Suturing is relatively time-consuming, however, and manually challenging. Good suture practice could reduce the incidence of complications and shorten suture time (11). Renal parenchymal bleeding is mainly controlled by blocking the renal pedicle blood vessels; however, prolonged room temperature renal ischemia would lead to renal warm ischemia and kidney function damage. Kijvikai *et al* (12) used ‘cold ischemia’ to occlude the renal pedicle. Although it clearly prolonged the occlusion time, the surgical procedure was complicated. Thompson *et al* (13) considered the optimal blocking time to be within 25 min following investigations into 362 cases. In the present study the renal artery blocking time was 20–30 min (average, 23 min). A follow-up of renal function post-operatively in all patients showed no obvious abnormality. The use of renal-rotation techniques could sufficiently expose the renal tumors for excision and suturing, ensure the closure of the section plane, therefore reducing warm ischemia time, protect residual renal function and prevent intraoperative complications, such as hemorrhage.

In the present study, which began in 2012, the renal-rotation technique was applied in a series of surgeries, including RPN of ventral side or inferior pole renal tumors. It has been demonstrated that this method can significantly increase the operating space, facilitate surgical procedures and shorten kidney warm ischemia time. Postoperative complications were found to be rare. The renal-rotation technique is, therefore, safe, feasible and worthy of wider clinical application.
